# Intravenous ketamine for benzodiazepine deprescription and withdrawal management in treatment-resistant depression: a preliminary report

**DOI:** 10.1038/s41386-023-01689-y

**Published:** 2023-08-02

**Authors:** Nicolas Garel, Kyle T. Greenway, Lê-Anh L. Dinh-Williams, Julien Thibault-Levesque, Didier Jutras-Aswad, Gustavo Turecki, Soham Rej, Stephane Richard-Devantoy

**Affiliations:** 1 Department of Psychiatry, Faculty of Medicine, McGill University, Montréal, QC Canada; 2 Lady Davis Institute, Jewish General Hospital, Montréal, QC Canada; 3 Research Centre, Centre Hospitalier de l’Université de Montréal (CRCHUM), Montréal, QC Canada; 4 Department of Psychiatry and Addictology, Faculty of Medicine, Université de Montréal, Montréal, QC Canada; 5 Douglas Mental Health University Institute, McGill Group for Suicide Studies, Montréal, QC H4H 1R3 Canada; 6 McGill Meditation and Mind-Body Medicine Research Clinic and Geri-PARTy Research Group, Lady Davis Research Institute and Jewish General Hospital, Montreal, QC Canada

**Keywords:** Outcomes research, Pharmacology

## Abstract

We present the first evidence that sub-anesthetic ketamine infusions for treatment resistant depression (TRD) may facilitate deprescription of long-term benzodiazepine/z-drugs (BZDRs). Long-term BZDR prescriptions are potentially harmful yet common, partly because of challenging withdrawal symptoms. Few pharmacological interventions have evidence for facilitating BZDR discontinuation, and none in patients actively suffering from TRD. In this ambi-directional cohort study, discontinuation of long-term (>6 month) BZDRs was attempted in 22 patients with severe unipolar or bipolar TRD receiving a course of six subanesthetic ketamine infusions over four weeks. We investigated the rates of successful BZDRs deprescription, trajectories of acute psychological withdrawal symptoms, and subsequent BZDRs abstinence during a mean follow-up of 1 year (primary outcome). Clinically significant deteriorations in depression, anxiety, sleep, and/or suicidality during the acute BZDR discontinuation phase were measured by repeated standardized scales and analyzed by latent growth curve models and percent correct classification analysis. Of the 22 eligible patients, all enrolled in this study and 91% (20/22) successfully discontinued all BZDRs by the end of the 4-week intervention, confirmed by urinary analyses. Less than 25% of discontinuers experienced any significant worsening of anxiety, depression, sleep difficulties, or suicidality during treatment. During follow-up (mean [range] duration, 12 [3–24] months), 64% (14/22) of patients remained abstinent from any BZDRs. These preliminary results suggest that ketamine infusions for TRD may facilitate the deprescription of BZDRs, even in patients with active depressive symptoms and significant comorbidity. Further investigation is warranted into this potential novel application of ketamine.

## Introduction

Approximately 30–50% of patients with depression are prescribed benzodiazepines and/or z-drugs (also known as Benzodiazepines and Related Drugs (BZDRs)) at some point during their illness [1]. Although international depression guidelines generally recommend only short-term BZDR use [2], chronic use eventually arises in 10–15% of patients with depression – particularly those with treatment resistant depression (TRD) [3, 4]. Long-term BZDR use has been linked to increased risks of falls and motor-vehicle accidents, cognitive impairment, suicide, and drug overdose mortality [5–9]. Deprescribing BZDRs may therefore yield benefits, in appropriate patients, but is often clinically challenging due to common and distressing withdrawal symptoms like rebound anxiety, insomnia, and depressive symptoms including increased suicidality [6, 7, 10, 11]. Anticipation of distressing withdrawal symptoms is often cited by patients and physicians as a reason to not pursue BZDR discontinuation in patients who may benefit [12].

Psychological and physical BZDR withdrawal symptoms are thought to arise from reduced GABAergic receptor responsiveness and increased expression of excitatory glutamatergic receptors [13–15]. Following BZDR cessation, withdrawal symptoms typically begin after 1-3 days, peak after 1-2 weeks, and resolve after about one month [5, 11], though they may potentially persist for months or years [16, 17]. Indeed, the term Post-Acute Withdrawal Syndrome has been conceptualized as such persistent symptoms occurring alongside significant psychological decline during or after benzodiazepine tapers [16, 18].

Few interventions have proven efficacy for facilitating BZDR discontinuation, particularly in patients with psychiatric illnesses like anxiety and depression that may increase vulnerability to withdrawal symptoms and their consequences [5, 6, 10, 19]. Clinical wisdom suggests that BZDR deprescription should generally only be considered in depressed patients who have achieved remission or at least stability [6]. To date, only one study has attempted BZDR deprescription in patients with active symptoms of depression [10, 20]. In that 10-week intervention, depressed chronic users of BZDRs were randomized to receive paroxetine or a placebo and switched to diazepam which was gradually tapered. The authors concluded that the addition of SSRI treatment to the valium-taper was of limited value [20, 21]. To our knowledge, no study has tested a behavioral and/or pharmacological intervention for BZDR deprescription in patients suffering from TRD.

In this study, we evaluated whether low-dose intravenous (IV) ketamine may facilitate long-term BZDR discontinuation in patients with active and severe TRD. Ketamine is a non-competitive inhibitor of glutamatergic NMDA receptors with GABA agonistic activities and evidence for rapid (<24 h) benefits against TRD [22]. Our ketamine-TRD service routinely attempts to discontinue all BZDRs given preliminary (albeit conflicting [23]) evidence that they may blunt ketamine’s antidepressant effects [22] and increase the rate of serious adverse events (according to post-marketing study of esketamine) [24], in addition to the potential long-term harms of BZDRs. For willing patients, we thus taper BZDRs such that last doses coincide within one or two days of the first ketamine treatment, based on the hypothesis that ketamine may reduce glutamatergic hypersensitivity – as suggested by preclinical and emerging clinical evidence for ketamine against alcohol withdrawal/addiction [15, 25] – and may mitigate common/severe BZDR acute withdrawal symptoms [15]. I.e., the rapid benefits of low-dose ketamine infusions against symptoms of depression [22], anxiety [26, 27], insomnia [28, 29], and suicidality may offset acute deteriorations caused by BZDR discontinuation [27, 28]. To explore these hypotheses, we examined group- and patient-level changes in these latter symptoms across six infusions of ketamine administered over one month, as well as subsequent BZDRs abstinence on follow-up, for patients in our service attempting BZDRs discontinuation.

## Methods

### Setting

This ambi-directional (i.e., containing both retrospective and prospective phases) single group cohort study occurred at the Ketamine Service of the Douglas Mental Health University Institute in Montreal, Quebec, Canada. Patients were referred from psychiatrists across the province of Quebec to this tertiary care service to receive ketamine for highly treatment-refractory unipolar and bipolar depression. The study was approved in November 2021 by the institutional review board of the Douglas Mental Health University Institute (#IUSMD-21-29) and individual written consent was obtained. Data collection was performed until August 2022. EQUATOR reporting guidelines were followed.

### Participants

Participants were recruited on an ongoing basis from the Douglas Ketamine service between November 2021 and May 2022. As is common in Montreal, participants were either primary French or English speaking. Inclusion criteria for the study were: 1) age >18, <75 years old; 2) received at least one ketamine infusion at the ketamine service for an episode of unipolar or bipolar depression diagnosed by a trained psychiatrist (according to DSM-5), which had not responded to at least two adequate trials of psychotropic drugs with level 1 evidence against bipolar and/or unipolar depression; 3) at least one long-term (>6 month) active BZDR prescription at the time of the first ketamine psychiatric evaluation; 4) no medication changes 2-weeks before and during treatment (except for BZDR reduction); and 5) provision of written informed consent. Otherwise, no exclusion criteria were utilized for this study, though all eligible patients had been accepted for ketamine treatments and thus met our service’s criteria, provided in the supplement information. Two noteworthy exclusion criteria are: current or recent history (i.e., in the past 12 months) of alcohol or cannabis abuse or dependence, and current or lifetime history of substance abuse or dependence (including all substances except for caffeine or nicotine), as defined by DSM-5 criteria [30].

A chronological, retrospective chart review of all patients of the ketamine-TRD service identified eligible patients who were initially contacted by telephone (by a research assistant) to introduce the study and to seek informed consent. Consenting patients were enrolled into the study’s prospective long-term follow-up phase and BZDR use-patterns were evaluated at multiple timepoints as detailed below.

### Intervention

#### Phase 1: Initial evaluation at the ketamine service and benzodiazepine gradual taper

All patients referred to the ketamine-TRD service underwent a 60–120 min psychiatric/medical evaluation, including laboratory investigations and an electrocardiogram, to determine their suitability for treatment by IV ketamine. After evaluation, accepted patients received one or two 30–60-min additional visits with the service’s clinicians before beginning ketamine for the purposes of psychological support, psychoeducation, and establishing rapport. Our service further ensures that all patients accepted for ketamine treatments receive one hour per week of psychological support or psychotherapy (e.g., with a psychologist, social worker, occupational therapist, counselor, etc.) during the acute ketamine treatment phase, typically with external clinicians, given evidence that ketamine can be psychologically destabilizing and that psychological treatments of TRD are often underutilized [31, 32]. The broad aim of these additional supports is to optimize the chances for acute and sustained antidepressant effects of ketamine.

BZDR discontinuation was discussed with all patients accepted for ketamine treatment based on evidence for harms as described above. Patients interested in stopping BZDRs were then offered to gradually decrease their dose by 10–25% per week before beginning their course of ketamine, aiming to take the last dose (i.e., 25% of the initial dose) within one or two days of the first treatment. All participants were taking intermediate-duration BZDRs, and thus withdrawal symptoms were expected to begin within 1–3 days of cessation, peak after 1–2 weeks, and resolve within one month [11], coinciding with the ketamine treatment phase. All patients were provided with the telephone number of the clinic’s nurse in case of issues arising before beginning ketamine treatments, including but not limited to BZDR withdrawal symptoms.

#### Phase 2: Ketamine infusions

The ketamine treatment consisted of six IV infusions (0.5 mg/kg of bodyweight) given over four weeks; twice weekly for two weeks then weekly for two weeks. Prior to every infusion, baseline vital signs were measured and a urinary drug screen plus a urine pregnancy test (if relevant) were administered. The urine drug screen was performed with PROFILE^®-^V drug testing cassette devices and a MEDTOXScan reader from MEDTOX Diagnostic Inc., a solid-phase immunoassay device, conforming with ISO 13485, capable of detecting 13 drugs including benzodiazepines. Pre-infusion questionnaires (including measures of mood, anxiety, suicidality, and sleep) were completed, and patients were also routinely asked if they had experienced any specific side-effects or adverse events from previous infusions. Any such events were recorded.

The patients received their infusions in a quiet room, laying on a bed. A vein was cannulated, and ketamine hydrochloride was diluted in 250 mL of normal saline by the treating team’s nurse, according to the patient’s weight and with verification by one other member of the treating team. In patients with a body mass index (BMI) greater than 30, ketamine doses were calculated based on a normalized BMI of 30, given that greater hemodynamic changes with a BMI above 30 have been observed [32]. Ketamine infusions were given in the presence of the nurse and a physician with ongoing assessments of patients’ physiological and mental status during the infusion, including respiratory status and cardiovascular functioning. Some patients were provided with music during their treatment sessions. Prior to discharge, patients were required to remain on premises for at least 1 h of observation after the infusion’s end. For emergent agitation or anxiety, midazolam (maximum dose 2.5 mg PO or IM) or another short-acting benzodiazepine were available (but not administered to any patients in the study sample).

Following the course of six infusions, the patients of our ketamine-TRD service are discharged to the care of their referring psychiatrists. Any decisions to restart BZDRs following the ketamine treatment course were made by patients and their healthcare providers, independent of our service.

### Outcomes and measures

Before initiating the study, we hypothesized that ketamine infusions in combination with a gradual taper would facilitate the deprescription of BZDRs in TRD patients by mitigating patient’s psychological deterioration and reducing common rebound anxiodepressive symptoms and insomnia [6, 17]. We set a priori continuation rules as described in the statistical analysis section.

### Sample characteristics

Sociodemographic and clinical characteristics (e.g., age, sex, psychiatric diagnosis, medical comorbidities) and prescribed medications were retrospectively compiled from the ketamine-service charts of all participants.

### Benzodiazepine and z-drug prescription information

BZDR prescription patterns (type, dosage, frequency, length of use) were collected using multiple sources of information at the initial evaluation, prior to every infusion, and at the end of the 4-week ketamine intervention. Sources included patient self-reports, referral documents, urine toxicology results, and the current prescriptions detailed in the *Dossier Santé Quebec* (DSQ). The DSQ is a secure provincial communication platform that facilitates timely sharing of health information between authorized organizations, physicians, and stakeholders, that collects and stores diverse health information on Quebec patients including active and past prescriptions. The DSQ is thus a reliable way to verify current and past prescriptions of a given patient.

Post-treatment BZDR use was obtained by contacting participants by telephone every 3–6 months post-treatment using a timeline follow-back approach (TLFB) [33], and by the provincial prescription database. The TLFB approach is a calendar–based form in which people provide retrospective estimates of their daily drug/medication consumption over a specified period of time [33]. Memory aids are used to enhance recall. The TLFB method has been extensively evaluated with a wide range of clinical populations and was chosen by the American Psychiatric Association as meeting criteria for inclusion in their Handbook of Psychiatric Measures [34]. Although less objective than urinary toxicology, the combination of self-report TLFB and provincial registry data would only theoretically miss illicit BZDR use, which was judged as unlikely for this population given that they had no significant histories of substance use disorders and were actively followed by prescribers who had previously prescribed them BZDRs. The study entry date of participants, determined by their ketamine treatment dates, dictated the length of follow-up and the number of post-treatment assessments. We used the following dose equivalencies for benzodiazepines, based on the most recent scientific evidence [35] : 15 mg of oxazepam equivalent to 5 mg of diazepam, 1 mg of lorazepam, 0.5 mg of clonazepam, and 0.5 mg of alprazolam. Z-drugs doses were not converted to benzodiazepine equivalence because of the inconsistencies in the literature, and thus were not used in the calculation of mean diazepam doses.

### Definition of abstinence

A variety of BZDR abstinence/discontinuation outcomes have been used in past research, including in depressed populations [20, 21]. We chose the percentage of complete abstinence (*no* active BZDRs use) at the end of the ketamine intervention and on follow-up as our pre-specified primary outcome, as detailed in the study protocol submitted to the Douglas Mental Health Ethical Review Board in June 2021 prior to data collection. This stringent definition reflects the service’s aim of total BZDR discontinuation, when possible, in order to optimize ketamine response [22]. There is no evidence, to our knowledge, indicating a dose-response interaction of BZDRs on the antidepressant response of ketamine.

### Psychological withdrawal outcomes

The secondary outcomes of this study were the clinical trajectories of common withdrawal symptoms observed in BZDRs discontinuation – depression, anxiety, sleep, and suicidality [6, 11, 17] – which we hypothesized would not significantly worsen despite the ketamine treatment process overlapping with the acute phase of BZDRs withdrawal.

For depressive symptoms, we utilized the Beck Depressive Inventory II (BDI-II) [36], a 21-item self-report scale with higher scores indicating more severe depressive symptomatology. Each item is scored on a 4-point Likert scale (total score range: 0–63) [36]. The BDI-II shows high internal consistency and test-retest reliability, reflects a broad range of depressive symptoms, and has been extensively utilized in clinical and research settings [37].

Current anxiety symptoms were measured by the State Trait Anxiety Inventory (STAI-A) [38], state sub-scale, which has 20 items rated on a 4-point scale (total score range: 20–80) with higher scores indicating greater anxiety [38]. Considerable evidence attests to the construct and concurrent validity of the scale, and its high test-retest reliability [39].

Sleep was assessed by the Leeds Sleep Evaluation Questionnaire (LSEQ), a scale initially designed to assess changes in sleep quality over the course of a psychopharmacological interventions [40, 41]. It contains 10 self-rated 100-mm-line analog questions (score ranges from 0 to 100) concerning versus aspects of sleep: getting to sleep, quality of sleep, awakening from sleep, and behaviors following wakefulness. Lower scores indicate more sleep difficulties and impairment. The LSEQ is one of the most commonly use sleep evaluation questionnaires in clinical settings, has high validity, and is sensitive to change [41, 42]. As the LSEQ assesses treatment-related changes in sleep quality, it was not administered at the first treatment, and thus the second ketamine treatment was utilized as the baseline value in all analyses.

Suicidality was assessed by the current-moment Beck Scale for Suicide Ideation (SSI), a widely used instrument to assess suicidality [43]. The SSI contains 19 items measuring severity of actual suicidal wishes and plans, with higher scores indicating a higher level of suicidal ideation (scores range from 0 to 38) [43]. The most sensitive cut-off for high versus low risk of suicide is >2, according to multiple studies [44].

For Francophone participants, we used the validated French versions of the BDI-II [45], STAI [46], LSEQ [42], and SSI [47].

### Subjective impressions of the intervention

Many patients in this study had made previous, unsuccessful attempts to discontinue BZDRs. As such, their feedback was elicited regarding the potential utility of ketamine using a brief questionnaire administered at follow-up as follows: “Please indicate, on a scale of 0–4, to what extend you agree with the following statement: “The ketamine intervention was helpful in stopping my prescription of <drug name>”.” Responses were given on a 5-point Likert scale (strongly disagree = 0, disagree = 1, neutral = 2, agree = 3, strongly agree = 4). Patients were also asked in an open-ended fashion to describe why the ketamine treatment was helpful or not helpful for discontinuing BZDRs, the results of which were thematically classified by the study team.

### Tolerability and drop-out

Adverse events and proportion of patients discontinuing the ketamine treatment for benzodiazepine withdrawal tolerability related reasons were recorded.

### Statistical analyses

We ran a pilot multi-method longitudinal investigation including both group- and person-level analysis methods. To determine if a clinical trial formally evaluating ketamine as an intervention for BZDRs deprescription is warranted, we set a priori continuation rules based on the only previous study on benzodiazepine discontinuation in depressed patients [20, 21]. For abstinence outcomes: 1) >65% of participants will be categorized as successful discontinuers (BZDR-abstinent as evidenced by self-report and urinary evaluation) by the end of the ketamine treatment; and 2) during follow-up, >30% of participants will be categorized as successful discontinuers (BZDRs-abstinent as evidenced by self-report). For withdrawal symptoms: 1) <40% of participants will show reliable clinical deteriorations in depression, anxiety, suicidality, and/or sleep; and 2) BZDR discontinuation will not lead to serious negative consequences (unexpected, clearly trial- or treatment-related serious adverse reaction) and/or significant treatment drop-out.

### Benzodiazepine abstinence

Patients who successfully discontinued all BZDRs and remained abstinent throughout follow-up were categorized as “abstinent”. Patients who never successfully discontinued all BZDRs by the end of the 4-week ketamine treatment protocol were categorized as “never abstinent”, and the remainder who successfully discontinued all BZDRs by the end of the 4-week ketamine treatment, but who restarted their BZDRs medication during follow-up were categorized as “restarted”. Descriptive statistics of clinical characteristics were calculated according to these abstinence outcomes. Additionally, we conducted a Kaplan-Meier survival analysis using the ‘survival’ package in R-4.2.3 to examine the rate, timing, and prediction of restarting BZDRs.

### Psychological withdrawal symptoms

For psychological withdrawal symptoms during the ketamine treatment course, we first examined intra-individual changes in withdrawal symptoms with latent growth curve (LGM) models using restricted maximum likelihood estimation of mixed-effects models. This approach performs well with small sample sizes to address bias in standard error estimates and inflated operating type I error rates [48]. Latent mixed-effects modeling was conducted with lmer() function from the lme4 package [49], in combination with lmerTest package [50], as implemented in R-4.2.3. We created latent growth curve models for each symptom using a stepped approach consistent with Bollen and Curran (see Supplement for details) [51].

Additionally, we conducted complementary percent correct classification (PCC) analyses, also known as person-centered effect sizes [52], as there is increasing recognition that statistical inferences drawn from groups of individuals may not accurately describe the individuals themselves [52]. Using the PCC approach, we examined how many patients matched the hypothesized benefits of ketamine in the management of BZDRs withdrawal – i.e., no reliable deteriorations in depression, anxiety, sleep, and suicidal ideation at subsequent treatment sessions (session 2, 3, 4, 5, or 6 vs. session 1).

Reliable change (RC) indices were calculated for each patient to determine whether they experienced changes in any of the four symptom dimensions that were statistically reliable and clinically significant, using the Leeds RC indicator tool [53]. Calculation of RC requires means and standard deviations (SDs) of clinical and comparison norms, in addition to scale reliability estimates. We used the following coefficient alphas for each scale: 0.92 (BDI-II) [54], 0.94 (STAI) [39], 0.84 (SSI) [43], and 0.84 (LSEQ) [55]. Following the statistical approach of Jacobson and Truax [56], individuals experiencing any reliable deterioration at a subsequent ketamine treatment (sessions 2, 3, 4, 5, or 6), relative to their baseline at the initial ketamine treatment (session 1), were classified as “deteriorated” in that symptom dimension regardless of whether they also experienced reliable improvements at any other point. Patients experiencing no reliable deteriorations were then classified as either overall “improved” (i.e., a reliable improvement at the session 6 relative to session 1), or “no change” (no reliable deterioration or improvement as defined above). In other words, patients experiencing any reliable deterioration were classified as deteriorated, whereas only those experiencing a reliable improvement at session 6 and no prior deteriorations were classified as improved.

## Results

### Clinical characteristics and demographics

**Fig. 1:**
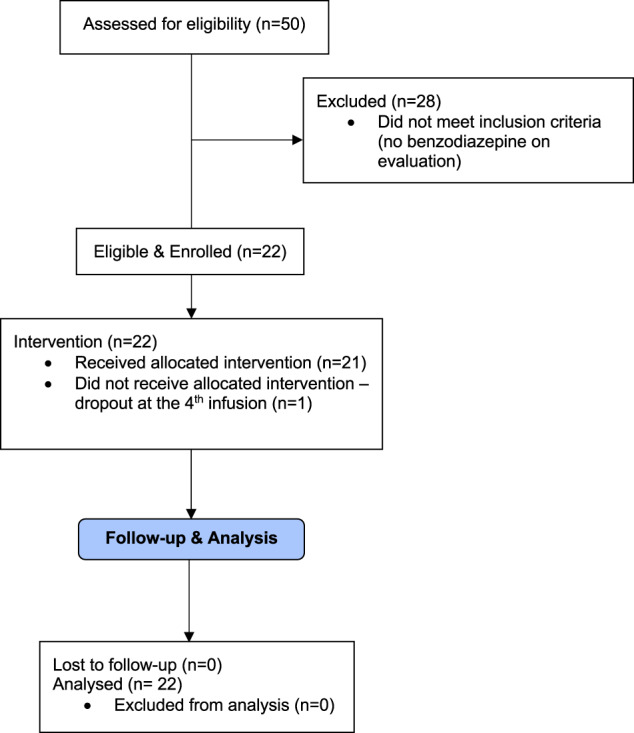
Consort diagram.

Of the 50 TRD patients treated by our ketamine service between July 2019 and February 2022, 44% (22/50) were chronic (>6 month) BZDR users on evaluation. All 22 chronic BZDRs users satisfied other inclusion/exclusion criteria and were approached for enrollment, with 100% (22/22) consenting to participate (Fig. 1). 64% were female; mean [range] age, 49 [23–69] years; 95% were Caucasian. All patients had severe TRD, unipolar or bipolar, with a mean baseline BDI-II score of 36.6 (SD = 12.6). Significant suicidality at baseline was present in 82% of the sample (SSI ≥ 2) with an average SSI score of 10.5 (SD = 9.5). Fifty-nine percent of patients were diagnosed with a comorbid anxiety disorder (*n* = 13) and 45% with a personality disorder (*n* = 10). Twenty-three percent were suffering from obstructive sleep apnea (*n* = 5). Regarding BZDR prescriptions, 64% (*n* = 14) were treated with only benzodiazepines, 18% with only z-drugs (*n* = 4), and 18% with both (*n* = 4). Benzodiazepines were reported to have been prescribed for comorbid anxiety disorders and/or for anxious distress associated with TRD, whereas Z-drugs were reportedly prescribed for insomnia. Baseline mean (SD) diazepam dose-equivalents (excluding z-drugs) and exposure duration were 15.6 (12.9) mg/day and 3.9 (4.8) years. Most patients (55%; *n* = 12) reported one or more past unsuccessful attempts at discontinuing chronic BZDRs, due to uncomplicated withdrawal symptoms and/or the unmasking of original targeted symptoms. No patients reported past discontinuation attempts with complicated or severe adverse events such as seizures or hospitalizations. Clinical characteristics and demographics are detailed in Table 1.

**Table 1:** Patient demographic and clinical characteristics at baseline, categorized by follow-up outcomes.

			BZDR outcomes categories during follow-up		
		Total sample *n* = 22	Abstinent *n* = 14	Restarted *n* = 6	Never abstinent *n* = 2
Gender	Female no. (%)	14 (64)	8 (57)	4 (67)	2 (100)
	Male no. (%)	8 (36)	6 (43)	2 (33)	0
Age (years)	M (SD)	49 (13)	47.6 (14.7)	50.0 (9.9)	58 (1.4)
Ethnicity (Caucasian)	No. (%)	21 (95)	13 (93)	6 (100)	2 (100)
Education (college)	No. (%)	16 (73)	11 (79)	4 (67)	1 (50)
Duration of BZDR prescription (years)	M (SD)	3.9 (4.8)	4.3 (5.7)	2.3 (1.5)	6.0 (5.6)
	Range	0.5–23.0	0.5–23.0	1.0–5.0	2.0–10.0
Dosage in diazepam equivalence (mg/day)	M (SD)	15.6 (12.9)	17.3 (12.9)	12.0 (5.7)	40.0 (NA)
Days of use (per week)	M (SD)	6.7 (0.9)	6.6 (1.1)	7.0 (0)	7.0 (0)
BZDR category	Clonazepam no. (%)	13 (59)	10 (71)	2 (33)	1 (50)
	Lorazepam no. (%)	5 (23)	3 (21)	2 (33)	0
	Alprazolam no. (%)	1 (5)	0	1 (17)	0
	Z-drugs no. (%)	8 (36)	4 (29)	3 (50)	1 (50)
Combination of two sedative/hypnotics	No. (%)	6 (27)	4 (29)	2 (33)	0
Length of gradual taper pre-ketamine (weeks)	M (SD)	6.2 (3.8)	6.1 (3.4)	5.1 (4.0)	0
	Range	0–12	2–8	4–12	0
Length of follow-up post-treatment (weeks)	M (SD)	52 (32.4)	51.2 (33.2)	66.6 (24.6)	25.5 (19.1)
	Range	12–110	12–110	24–98	12–39
Past failed attempts at BZDR discontinuation	M (SD)	1.7 (4.3)	2.2 (4.0)	0.8 (4.0)	0.5 (NA)
Type of mood disorder	MDD no. (%)	17 (77)	10 (71)	5 (83)	2 (100)
	BD no. (%)	5 (23)	4 (29)	1 (17)	0
Psychiatric comorbidities	Anxiety^a^ no. (%)	13 (59)	8 (57)	5 (83)	0
	PTSD no. (%)	6 (27)	4 (29)	2 (33)	0
	ADHD no. (%)	4 (18)	2 (14)	2 (33)	0
	PD no. (%)	10 (45)	6 (42.9)	2 (33)	2 (100)
	Other no. (%)	13 (59)	8 (57)	2 (33)	2 (100)
Non-BZDR Psychotropes	M (SD)	2.7 (1.5)	2.7 (1.7)	3.0 (0.3)	2.0 (1.4)
Antidepressant	No. (%)	20 (91)	13 (93)	6 (100)	1 (50)
Antipsychotic	No. (%)	11 (50)	6 (43)	3 (50)	2 (100)
Mood stabilizer	No. (%)	8 (36)	6 (43)	1 (17)	1 (50)
Psychostimulant	No. (%)	6 (27)	3 (21)	3 (50)	0
Chronic physical conditions	No. (%)	17 (77)	11 (79)	5 (83)	1 (50)
OSA	No. (%)	5 (23)	4 (29)	0	1 (50)
Baseline scale scores					
BDI-II	M (SD)	36.6 (12.6)	36.6 (14.3)	35.3 (9.4)	40.5 (14.8)
STAI-A	M (SD)	58.5 (11.7)	55.8 (12.3)	59.3 (7.3)	75.0 (4.2)
SSI	M (SD)	10.5 (9.5)	11.9 (10.6)	7.1 (8.2)	9.5 (9.2)
LSEQ	M (SD)	40.9 (10.4)	44.0 (10.4)	36.4 (11.2)	39.4 (10.8)

*ADHD* Attention deficit/hyperactivity disorder, *BDI-II* Beck Depressive Inventory II, *BZDR* benzodiazepine and/or z-drugs, *LSEQ* Leeds Sleep Evaluation Questionnaire, *OSA* Obstructive sleep apnea, *PD* Personality disorder, *PTSD* Post-traumatic stress disorder, *SSI* Scale for Suicide Ideation (current), *STAI-A* State-Trait-Anxiety-Inventory (state).

^a^Anxiety disorders includes Social Anxiety Disorder, Generalized Anxiety Disorder, Panic Disorder and Agoraphobia.

### Primary outcome: BZDR discontinuation

All patients with BZDR prescriptions on evaluation agreed to receive six infusions of ketamine and attempt BZDR discontinuation. Twenty-one patients (95%) completed the ketamine intervention per protocol. Only one client did not complete all ketamine sessions and discontinued after four infusions. At the end of the 4-week intervention, 20 patients (91%) had successfully stopped all BZDRs as confirmed by urine testing, self-report, and the centralized provincial prescription databank. During the subsequent follow-up period of mean [range] 12 months [3–24], 14 patients (64%) remained BZDR-free. The other six discontinuers reinitiated BZDRs and were thus classified as “restarted”, albeit with a mean [range] 53% [0–85] decrease in daily dose. Several primary reasons were reported by these six patients for restarting BZDRs: four patients reported an exacerbation of insomnia/anxiety symptoms (with stable mood symptoms), one reported a depressive episode relapse, and one reported restarting BZDRs to mitigate the side effects of initiating a new antidepressant medication.

**Fig. 2:**
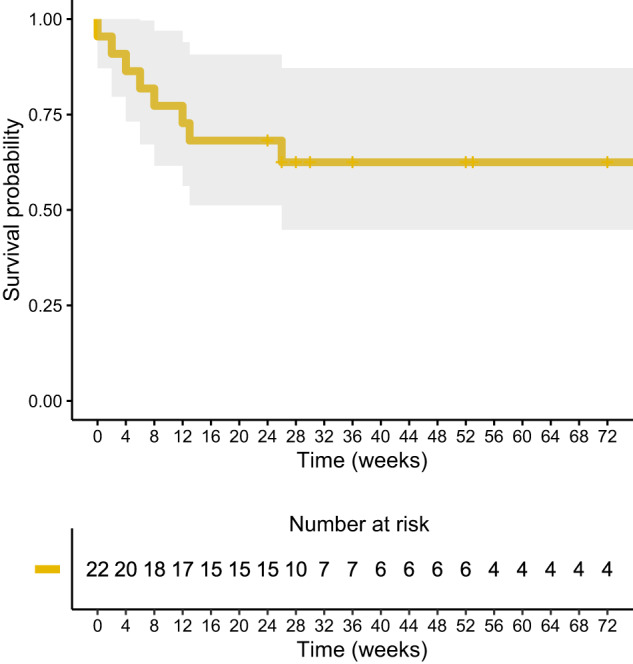
Unadjusted Kaplan–Meier estimates of BZDR restarting for successful discontinuers after the ketamine intervention. Kaplan–Meier survival curve showing time to restarting BZDRs, in weeks, with an estimated cumulative survival rate of 68% (95% CI: 0.51–0.91). The numbers below the Kaplan–Meier curves represent the numbers of patients followed up and the numbers censored at each timepoint.

Figure 2 presents the survival curve for the full cohort. The mean survival time was 72 weeks, with the probability of abstinence decreasing gradually post-treatment until levelling off at six months, yielding a cumulative survival rate of 68% (95% CI: 0.51–0.91).

### Secondary outcomes: withdrawal symptoms

Overall, significant pre-post improvements in depression, anxiety, suicidality, but not sleep quality were observed with group-level LGM analyses. On average, participants reported significant decreases in BDI-II (β = −2.57, SE = 0.36, t(107) = −7.19, *p* < 0.001), STAI-A (β = −1.81, SE = 0.36, t(107) = −5.09, *p* < 0.001), and SSI (β = −1.16, SE = 0.26, t(104) = −4.39, *p* < 0.001) scores with each ketamine treatment, but not LSEQ scores (β = 0.71, SE = 0.61, t(86) = 1.15, *p* = 0.251) (see supplement for more information on LGM results and model fit). This corresponds to meaningful overall decreases in depressive symptoms (baseline mean BDI-II score 36.6 (SD = 12.6), posttreatment mean BDI-II score 23.1 (SD = 12.7)), anxiety (baseline mean STAI-A score 58.5 (SD = 11.8), posttreatment mean STAI-A score 46.9 (SD = 12.7)), and suicidality (baseline mean SSI score 10.5 (SD = 9.5), posttreatment mean SSI 4.0 (SD = 5.9)), without significant changes in subjective sleep quality (baseline mean LSEQ score 40.9 (SD = 10.4), posttreatment LSEQ score 42.7 (SD = 12.4).

**Fig. 3:**
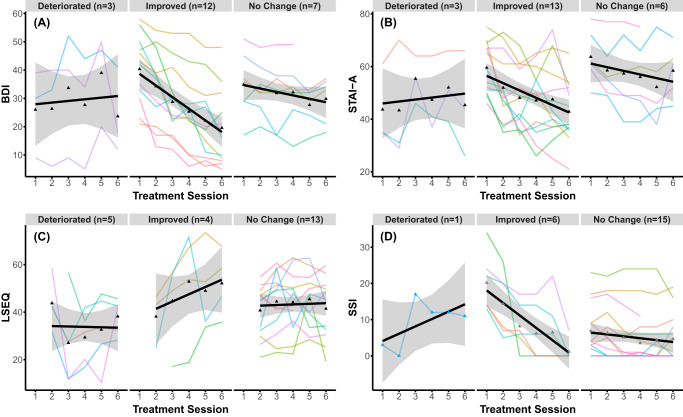
Symptom trajectories during treatment and acute withdrawal. Illustration of raw clinical scores (Y axis) for (**A**) depression (BDI-II), (**B**) anxiety (STAI-A), (**C**) sleep (LSEQ), and (**D**) suicidality (SSI) over each ketamine treatment session (X axis) for each subgroup of treatment responses. The black line represents estimated changes in symptoms over time for each subgroup with the mean at each treatment session (triangular dot) and standard error of the mean (grey). Each subject’s raw clinical trajectory is displayed as colored lines. Individual patients grouped as deteriorated (any significant deterioration), improved (significant improvement without any significant deterioration), or no change (no significant deterioration or improvement). BDI-II Beck depression Inventory, STAI-A State-Trait-Anxiety-Inventory (State), LSEQ Leeds Sleep Evaluation Questionnaire, SSI Scale for suicide ideation (current).

PCC analyses revealed that the large majority of participants did not experience any significant deterioration at any treatment visit, relative to baseline, in depression (86%) (Fig. 3A), anxiety (86%) (Fig. 3B), sleep (77%) (Fig. 3C), or suicidality (96%) (Fig. 3D) (see Supplementary Table S1 in supplement for more information on PCC analyses). PCC analyses largely converged with LGM group trajectories. At the end of treatment, more than half of patients had reliable improvements in depression (55%; *n* = 12) and anxiety (59%; *n* = 13), versus approximately a quarter for sleep (18%, *n* = 4), and suicidality (27%, *n* = 6). Of those experiencing any reliable deterioration at any treatment timepoint, most had returned to baseline or had reliably improved at the final infusion, in terms of depression (2/3), anxiety (2/3), and sleep (4/5), but not suicidality (0/1) (see Supplementary Table S2 in supplement for raw scores).

### Subjective appreciation

On average, our sample of 22 long-term BZDR users patients reported two prior unsuccessful attempts at discontinuing BZDRs, suggesting some pre-existing motivation to decrease or stop BZDRs prior to the ketamine treatment process. After the intervention, 12 out of 22 clients (54.5%) rated their agreement with the statement that ketamine had been helpful for BZDRs discontinuation as 4 of a maximum 4 (“strongly agree”). Only one client reported 0 of 4 (“strongly disagree”) (Table 2).

**Table 2:** Subjective appreciation outcomes of ketamine therapeutic impact on benzodiazepine discontinuation based on response to statement: “The ketamine intervention was helpful in stopping my prescription of <drug name>”.

Likert scale results	Total sample no. (%)
0/4 – “strongly disagree”	1 (5)
1/4 – “disagree”	0
2/4 – “neither agree nor disagree”	2 (9)
3/4 – “agree”	7 (32)
4/4 – “strongly agree”	12 (55)

Patients gave convergent reasons for why the ketamine treatment process had been helpful for discontinuing BZDRs: 1) decreased depressive symptomatology; 2) decreased anxiety levels; 3) reduced withdrawal symptoms (including sleep impairment); 4) motivation to potentially increase the antidepressive effects of ketamine; and 5) benefits from support received throughout the treatment process. These reports may reflect some desirability bias.

### Tolerability

Only one patient did not complete the study protocol due to poor tolerability of ketamine’s psychoactive effects resulting in discontinuation of treatment after four infusions. This patient was one of the two patients who did not discontinue BZDRs. Outside of the psychological symptoms analyzed in this study, three patients complained of physical withdrawal symptoms during the first week of the treatment: muscle spasms, tinnitus, and muscle pain/stiffness. All were mild and transient. Additionally, four participants reported significant desires to use their prescribed BZDR medications during the first two weeks of the study, while receiving bi-weekly ketamine infusions, due to transient increases in anxiety or insomnia.

## Discussion

In this cohort study, we report treatment outcomes and follow-up data of 22 severe TRD participants attempting chronic-BZDR discontinuation with a course of six ketamine infusions. Twenty-one participants completed all six treatments of the 4-week ketamine protocol and, using the stringent criteria of total abstinence, 91% (20/22) successfully discontinued all BZDRs by its end, as confirmed by several means including urine toxicology. Sixty-four percent (14/22) of patients remained abstinent after an average naturalistic follow-up of one year, as per self-report and the provincial prescription database, with the risk of restarting BZDRs stabilizing after six months.

Only a minority (≤25%) of participants experienced clinically significant deterioration in depression, anxiety, sleep, or suicidality at any timepoint during the treatment process by PCC analysis. Indeed, group-level analyses revealed overall improvements (all *p* < 0.001), except for sleep quality. These results contrast with typical rates of BZDR withdrawal symptoms occurring in 40–100% of discontinuers, even with gradual tapering, most commonly in the days-weeks following the last quarter of the original dose [17, 20, 57].

Chronic BZDR deprescription is a complex endeavor for both clinicians and patients, and is even more challenging in patients actively suffering from psychiatric illness like depression [16, 20, 21]. To our knowledge, this is the first report of a successful intervention to deprescribe BZDRs in chronic users during an acute episode of TRD. Only one other study of patients with active depression has been conducted, to our knowledge, finding 6-month and 24-month abstinence rates of 32% and 14% following a 10-week intervention combining paroxetine and diazepam [20].

There is evidence to suggest that rational deprescription of BZDRs may be of particular value in TRD populations despite inherent challenges. In our real-world sample of severe unipolar and bipolar TRD patients, nearly 50% received long-term BZDR, with an elevated average daily dose of 15.6 mg (diazepam equivalent). Indeed, similarly elevated rates of benzodiazepine prescription have been found in other studies of ketamine [4, 58], congruent with the two to threefold increased risk of sedative use disorder in TRD [59]. Preliminary evidence further suggests a potential correlation between BZDRs and more severe/chronic illness courses in depression (although the causality of this link has yet to be established) [60]. TRD populations are also at higher risk than general and non-resistant depressed populations for polypharmacy and medical comorbidities like OSA [61], which may increase the potential harms of BZDRs [62]. Indeed, 23% of our study sample had a diagnosis of OSA and patients, on average, received 2.7 psychotropic medications (excluding BZDRs and ketamine). Lastly, TRD is associated with greater levels of cognitive impairment than non-resistant depression, especially executive functioning, which has been linked to social and occupational dysfunction [63]. The potential for long-term cognitive harms of BZDRs further suggests therapeutic value in rational deprescription interventions [64].

As our results suggest, a course of sub-anesthetic ketamine treatments for mood disorders may provide a unique window of opportunity for making challenging medication changes, especially discontinuing BZDRs, due to several complementary mechanisms. Ketamine’s benefits may generally mitigate associated clinical deteriorations by rapidly alleviating common and dangerous depressive symptoms, including suicidality [22]. Pre-clinical evidence also suggests that ketamine may have direct benefits against the withdrawal states of GABAergic psychotropics (including common emotional withdrawal symptoms) [15], which have been associated with elevated NDMA receptor density in several cerebrocortical regions [13, 65]. Indeed, preliminary clinical evidence has found benefits of ketamine in severe alcohol withdrawal and refractory seizures [25], as well as in acute and severe benzodiazepine withdrawal (in one recent benzodiazepine use disorder case reports) [66], putatively due to neurotrophic and modulatory effects of ketamine on neuroexcitatory NMDA stimulation. Those findings suggest that our results in TRD may also hold relevance for patients with benzodiazepine use disorder, though the higher medical risks for such populations would likely necessitate closer monitoring such as is available in inpatient settings. Finally, the novelty and public interest in ketamine as an antidepressant may translate into enhanced motivation for patients to undertake the often-challenging process of discontinuing long-term medications, in order to increase their chance of responding to a treatment often seen as “last-line”. Indeed, at our ketamine-TRD service, 100% of patients agreed to attempt BZDR discontinuation.

The interpretation of this preliminary report is limited by its small sample size, lack of a control group, varying length of follow-up, inability to examine the impact of sex on outcomes of interest, and, most importantly, the lack of standardized scales of BZDRs withdrawal. Despite those limitations, we present the first quantitative and qualitative evidence that ketamine may facilitate discontinuation of chronic BZDRs in a particularly challenging real-world population of severe TRD patients with substantial comorbidity and suicidality. Our preliminary results of high rates of successful BZDRs discontinuation and low rates of significant psychological withdrawal symptoms may reflect ketamine’s benefits in depression and/or in BZDRs withdrawal states, or more non-specific expectancy factors. Future research, including controlled trials that rigorously assess physiological as well as psychological withdrawal symptoms, for this potential application of ketamine, is warranted.

### Supplementary information

Supplemental material
