# Supplementary material for: Intravenous ketamine for benzodiazepine deprescription and withdrawal management in treatment-resistant depression: a preliminary report
Source: Neuropsychopharmacology. 2023 Aug 2;48(12):1769–77. doi: 10.1038/s41386-023-01689-y (PMC10579413; doi:10.1038/s41386-023-01689-y)
Supplement: Supplementary file 1 — Supplemental material [file 41386_2023_1689_MOESM1_ESM.doc]

**SUPPLEMENTARY MATERIAL**

1. **Detailed exclusion criteria of the Douglas Mental Health University Institute Ketamine service**
2. **Detailed complementary statistical method and results**
   1. **table S1: Psychological withdrawal symptoms trajectory outcomes according to discontinuation results on long-term follow-up**
   2. **table S2: Raw Scores and Reliable Change of Patients with Deteriorations During Treatment**
3. **Exclusion criteria**

1) Previous non-response to ketamine in the current major depressive episode; 2) Known intellectual deficiency; 3) Prior or current substance abuse or dependence (except for caffeine or nicotine dependence) and/or recent history (last 12 months) of alcohol or cannabis abuse or dependence, as defined by DSM-V criteria; 4) Acute psychotic symptoms, as judged by the initial clinical interview or reported by referring clinicians; 5) Known risk factors for intracranial hemorrhage, including previous significant trauma, known aneurysm, or previous neurosurgery; 6) Pregnant, lactating, or of childbearing potential unwilling to use highly effective contraception; or 7) A clinical medical finding that is unstable or that, in the opinion of the treating clinician(s), would be negatively affected by, or would affect, ketamine (e.g., liver function tests three times the upper normal limit at screening, uncontrolled hypertension, etc.).

1. **Detailed complementary results**

**Latent Growth Curve Analyses**

In a first step, we calculated an intercept-only model including the random effects of participants to provide a baseline comparison (model 0 – intercept only). Subsequently, we ran a fixed effects model with time during treatment (in weeks) entered as a fixed predictor of symptoms (model 1 – intercept model with level-1 predictor) and a random slope model with time as a random slope (model 2 – intercept model with level-1 predictor and random slope). We used the function r.squaredGLMM() of the MuMIn package in R [1] to estimate the variance explained by both fixed and random factors as a measure of effect size. This allowed examination of the average linear rate of changes, in a given symptom dimension, across the treatment period, and whether patient-specific trajectories deviated from the baseline model.

**BDI-II.** Preliminary analyses (M0) revealed that the Conditional R^2^ related to the random intercept of the participants was equal to 0.68, suggesting a large amount of variability in depressive symptoms between participants. Treatment (M1) had a significant negative relationship with depressive symptom severity, b = -2.57, SE = 0.36, t(107) = -7.19, p < .001, explaining an additional 9% of the variance in depressive symptoms (Conditional R^2^= 0.77). Specifically, for each week of ketamine treatment, depressive symptoms decreased on average by 2.57 points on the BDI. Adding the random slope of treatment (M3) significantly improved model fit relative to the fixed-effects and random intercept model (M.2.), χ^2^(2) = 31.32, p <.001, and explained an additional 10.5% of the variance in symptoms (Conditional R^2^= 0.88). This suggests that the rates of change during treatment significantly varied between participants.

**STAI-A.** Preliminary analyses (M0) revealed that the Conditional R^2^ related to the random intercept of the participants was equal to 0.74, suggesting that there is a large amount of variability in state anxiety (STAI) between participants. Treatment (M1) had a significant negative relationship with STAI, b = -1.81, SE = 0.36, t(107) = -5.09, p < .001, explaining an additional 4.4% of the variance in STAI (Conditional R^2^= 0.78). Specifically, for each week of ketamine treatment, STAI decreased on average by 1.16 points. Adding the random slope of treatment (M3) did not significantly improve model fit relative to the fixed-effects and random intercept model (M2), χ^2^(2) = 3.06, p =.22, suggesting that the rates of change in STAI during treatment did not significantly vary between participants.

**LSEQ.** Preliminary analyses (M0) revealed that the Conditional R^2^ related to the random intercept of the participants was equal to 0.58, suggesting that there is a large amount of variability in sleep (LSEQ) between participants. Treatment (M1) did not have a significant relationship with sleep, b = 0.71, SE = 0.61, t(86) = 1.15, p = .251, Conditional R^2^= 0.58. Moreover, adding the random slope of treatment (M3) did not significantly improve model fit relative to the fixed-effects and random intercept model (M2), χ^2^(2) = 1.46, p =.48, suggesting that the rates of change in sleep during treatment did not significantly vary between participants.

**SSI.** Preliminary analyses (M0) revealed that the Conditional R^2^ related to the random intercept of the participants was equal to 0.6, suggesting that there is a large amount of variability in suicidal ideation (SI) between participants. Treatment (M1) had a significant negative relationship with SI, b = -1.16, SE = 0.26, t(104) = -4.39, p < .001, explaining an additional 5.4% of the variance in suicidal ideation (Conditional R^2^= 0.65). Specifically, for each week of ketamine treatment, SI decreased on average by 1.16 points on the SSI. Adding the random slope of treatment (M3) significantly improved model fit relative to the fixed-effects and random intercept model (M2), χ^2^(2) = 36.56, p <.001, and explained an additional 17.6% of the variance in symptoms (Conditional R^2^= 0.83). This suggests that the rates of change in SI during treatment significantly varied between participants.

**Percent Correct Classification**

PCC analyses allowed us to examine how many of the 22 participants showed no reliable deteriorations in withdrawal symptoms across the five ketamine treatment sessions relative to baseline (session one except for sleep, which was session two). Analyses revealed that the large majority of patients matched the expected pattern for each withdrawal symptom (STable 1): 86.4 % for the BDI, 86.4% for STAI , 95.5% for SSI, and 77.3% for LSEQ.

For depression, only three patients (13.6%) experienced a reliable deterioration in symptoms. Of these: one patient experienced reliable and large deteriorations of symptoms by S4, S5, and S6 relative to baseline symptoms at S1; one patient had relatively mild levels of depressive symptoms at S1 (BDI = 9) which deteriorated by S5 and returned to normal (no RC) by S6; and one patient experienced primarily no changes in depressive symptoms during treatment but a reliable deterioration at S5, followed by reliable improvements at S6 relative to S1. For anxiety, three patient (13.6%) experienced a reliable deterioration in symptoms. Of these patients: one patient reliably deteriorated from Sessions 1 to 2, followed by no reliable changes for the rest of treatment; one patient deteriorated at S3 but reliably improved by S6; and one patient showed reliable deterioration in symptoms at S3, S5, and S6. Five patients (22.7%) experienced reliable deteriorations in sleep during treatment; however, four of these patients experienced reliable deteriorations primarily at S3 or S4, and only one patient experienced consistent deteriorations in sleep relative to baseline. Only one patient experienced a reliable deterioration in suicidal ideation; however, this increase in suicidality from baseline (SSI = 3) appeared at Session 3 (SSI = 17) and was followed by no reliable change in SSI for subsequent sessions S4 (SSI = 12), S5 (SSI = 12), and S6 (SSI = 11).

**STable 1. Psychological withdrawal symptom trajectories according to discontinuation results on long-term follow-up**

|  | **Abstinent**  **N (%)** | | | **Restarted / Never abstinent**  **N (%)** | | |
| --- | --- | --- | --- | --- | --- | --- |
|  | **Deteriorated** | **Improved** | **No change** | **Deteriorated** | **Improved** | **No change** |
| **BDI-II** | 2 (9.1) | 8 (36.4) | 4 (18.2) | 1 (4.5) | 4 (18.2) | 3 (13.6) |
| **STAI-A** | 2 (9.1) | 10 (45.5) | 2 (9.1) | 1 (4.5) | 3 (13.6) | 1 (4.5) |
| **LSEQ** | 3 (13.6) | 3 (13.6) | 8 (36.4) | 2 (9.1) | 1 (4.5) | 5 (22.7) |
| **SSI** | 0 (0) | 6 (27.3) | 8 (36.4) | 1 (4.5) | 0 (0) | 7 (31.8) |

Abbreviation : BDI-II: Beck depression Inventory-II; STAI-A: State-Trait-Anxiety-Inventory (State); LSEQ: LEEDs Sleep Evaluation Questionnaire; SSI: Scale for suicide ideation

**STable 2. Raw scores and reliable change of patients with deteriorations during treatment**

|  | **BDI-II Total** | | | | | | **Reliable Change** | | | | |
| --- | --- | --- | --- | --- | --- | --- | --- | --- | --- | --- | --- |
| **Participants** | **S1** | **S2** | **S3** | **S4** | **S5** | **S6** | **S2 vs. S1** | **S3 vs. S1** | **S4 vs. S1** | **S5 vs. S1** | **S6 vs. S1** |
| 1 | 30 | 33 | 52 | 44 | 47 | 41 | Nochange | Deteriorate | Deteriorate | Deteriorate | Deteriorate |
| 2 | 9 | 6 | 9 | 5 | 20 | 12 | Nochange | Nochange | Nochange | Deteriorate | Nochange |
| 3 | 39 | 40 | 40 | 34 | 50 | 18 | Nochange | Nochange | Nochange | Deteriorate | Improve |
|  | **STAI-A Total** | | | | | | **Reliable Change** | | | | |
|  | **S1** | **S2** | **S3** | **S4** | **S5** | **S6** | **S2 vs. S1** | **S3 vs. S1** | **S4 vs. S1** | **S5 vs. S1** | **S6 vs. S1** |
| 2 | 35 | 29 | 56 | 37 | 51 | 44 | Nochange | Deteriorate | Nochange | Deteriorate | Deteriorate |
| 4 | 61 | 70 | 64 | 64 | 66 | 66 | Deteriorate | Nochange | Nochange | Nochange | Nochange |
| 5 | 35 | 31 | 46 | 41 | 39 | 26 | Nochange | Deteriorate | Nochange | Nochange | Improve |
|  | **LSEQ Total** | | | | | | **Reliable Change** | | | | |
|  | S1 | **S2** | **S3** | **S4** | **S5** | **S6** | **S3 vs. S2** | **S4 vs. S2** | **S5 vs. S2** | **S6 vs. S2** | |
| 1 | NA | 31.79 | 11.89 | 16.84 | 26.63 | 28.21 | Deteriorate | Deteriorate | Nochange | Nochange | |
| 3 | NA | 58.42 | 11.79 | 20.11 | 10.32 | 42.11 | Deteriorate | Deteriorate | Deteriorate | Deteriorate | |
| 4 | NA | 42.15 | 27.59 | 31.52 | 33.67 | 32.41 | Deteriorate | Nochange | Nochange | Nochange | |
| 6 | NA | 56.67 | 56.67 | 36.36 | 47.66 | 45.64 | Nochange | Deteriorate | Nochange | Nochange | |
| 7 | NA | 42.53 | 27.22 | 41.9 | 44.68 | 42.53 | Deteriorate | Nochange | Nochange | Nochange | |
|  | **SSI Total** | | | | | | **Reliable Change** | | | | |
| **ID** | **S1** | **S2** | **S3** | **S4** | **S5** | **S6** | **S2 vs. S1** | **S3 vs. S1** | **S4 vs. S1** | **S5 vs. S1** | **S6 vs. S1** |
| 1 | 3 | 0 | 17 | 12 | 12 | 11 | Nochange | Deteriorate | Nochange | Nochange | Nochange |

Abbreviation: BDI-II: Beck depression Inventory-II; STAI-A: State-Trait-Anxiety-Inventory (State); LSEQ: Leeds Sleep Evaluation Questionnaire; SSI: Scale for suicide ideation; S = session number; NA = not available

**References**

1 Barton K. MuMIn: Multi-Model Inference (1.46.0). 2022.
